# The Dysphagia Outcome and Severity Scale (DOSS) and non-instrumental swallowing measures in amyotrophic lateral sclerosis

**DOI:** 10.1007/s10072-026-09076-3

**Published:** 2026-05-07

**Authors:** Sergio Motta, Giuseppe Quaremba, Lucia Aruta, Salvatore Allosso, Gianmaria Senerchia, Valentina Virginia Iuzzolino, Raffaele Dubbioso

**Affiliations:** 1 Department of Advanced Biomedical Sciences, University of Naples Federico II, Via P. Stazio, 8, Naples, 80123 Italy; 2 Department of Neuroscience, Reproductive and Odontostomatological Sciences, University of Naples Federico II, Naples, Italy

**Keywords:** Amyotrophic lateral sclerosis, Dysphagia, Swallowing disorders, Dysphagia outcome and severity scale, Swallowing assessment

## Abstract

**Purpose:**

To evaluate reliability of the Dysphagia Outcome and Severity Scale (DOSS) in Amyotrophic Lateral Sclerosis (ALS) patients, and to assess diagnostic accuracy of selected non-instrumental measures in defining swallowing safety in this population.

**Methods:**

One hundred and thirteen consecutive ALS patients underwent comprehensive dysphagia evaluation with fiberoptic endoscopic evaluation of swallowing (FEES) and were classified according to DOSS. Safe and unsafe swallowing were defined by DOSS levels 7–6 and 5–1, respectively. Patient-reported measures included ALS Functional Rating Scale-Revised swallow item (I-3) and Eating Assessment Tool-10 (EAT-10). Non-instrumental clinical measures were hyolaryngeal excursion, voluntary cough (VC), voice quality and reflexive cough/throat clearing (VRC), and maximum phonation time (MPT). Inter- and intra-rater reliability were assessed using weighted Cohen’s kappa and Fleiss’ kappa coefficients. Non-instrumental measures diagnostic performance was evaluated using receiver operating characteristic (ROC) curve analysis.

**Results:**

Twenty-six of 113 patients (23%) exhibited an unsafe swallowing. Inter- and intra-rater agreement for DOSS classification was excellent across raters. EAT-10 and a composite clinical index derived from VC, VRC, and MPT showed the highest diagnostic accuracy with area under the curve values of 0.790 and 0.832, respectively. Other non-instrumental measures demonstrated lower discriminative performance.

**Conclusions:**

The DOSS showed an excellent reliability when applied to FEES in patients with ALS, supporting its use as a functional classification tool with direct nutritional and management implications. Non-instrumental measures should be interpreted with caution and confined to a triage role rather than diagnostic decision-making, particularly in light of the rapid progression of dysphagia in ALS.

## Introduction

Swallowing disorders are highly prevalent and alarming in individuals with Amyotrophic Lateral Sclerosis (ALS), potentially leading to malnutrition, dehydration and aspiration-related bronchopulmonary complications, with possible fatal outcomes [1, 2]. Fiberoptic endoscopic evaluation of swallowing (FEES), part of a comprehensive dysphagia evaluation, is currently considered the gold standard alongside video-fluoroscopic swallow study (VFSS) [3].

To date, studies on dysphagia in ALS have mainly adopted impairment-based scales, as the Penetration Aspiration Scale [4]. Alternative scales reflecting on functional outcomes and nutritional implications received less attention, despite being validated in studies involving both VFSS and FEES. Among these, the Dysphagia Outcome and Severity Scale (DOSS) distinguishes nutritional status levels and personal autonomies with related dietary implications [5–7]. The DOSS incorporates functional impact of dysphagia and diet limitations related to food consistency, provides guidance regarding level of support/independence in oral intake and offers a person-centred dysphagia management perspective [6, 7].

The potential underestimation of aspiration risk for trial feeding emphasizes the need for non-instrumental measures that meet criteria for practical feasibility in clinical settings [8, 9]. ALS Functional Rating Scale-Revised (ALSFRS-R), introduced in 1999 to assess global functional status and daily activities in ALS, is routinely administered from the time of diagnosis [10]. Only two previous studies verified the reliability of ALSFRS-R as dysphagia screening tool, either via the bulbar subset [11] or also through responses to I-3 (“*swallow item*”) [12]. The well-known and widespread Eating Assessment Tool (EAT-10), primarily designed to assess health-related quality of life [13], has also been investigated for its predictive value in identifying severe dysphagia in ALS [11, 14].

Hyolaryngeal excursion during swallowing, quality of voluntary cough, vocal emission and onset of reflex cough and/or throat clearing after swallowing represented clinical features used as non-instrumental clinical measures (NICMs) for screening and clinical evaluation of heterogeneous dysphagias [15–19]. Moreover, several studies highlighted the relationship between phonatory duration reduction and severe swallowing disorders, suggesting the use of maximum phonation time as non-instrumental measure for screening dysphagia in ALS [20–24].

The primary objective of the present study was to assess DOSS validity and reliability in a population of newly diagnosed ALS patients attending a single tertiary care center. Secondly, we aimed to determine the reliability of non-instrumental measures in discriminating swallowing safety for screening purposes in these patients.

## Materials and methods

### Study design and participants

This observational cross-sectional design study was approved by the Institutional Review Board and conducted according to Helsinki Declaration on Ethical Principles for Medical Research Involving Human Participants. All patients consecutively referred to the Neuromuscular Diseases Service of the Department of Neurology at University “Federico II” and diagnosed with ALS according to the revised El Escorial Criteria between October 2022 and October 2024 were considered for inclusion. Subjects receiving exclusive artificial nutrition were excluded from the study. A total of 113 patients were enrolled after providing written informed consent.

### Applying comprehensive dysphagia evaluation to DOSS

Each patient underwent FEES as a part of a comprehensive dysphagia evaluation, performed by two clinicians who worked as a couple, specialized in ENT and with a minimum of 5 years of experience in FEES, one of whom with an in-depth knowledge and experience in DOSS, both blinded to non-instrumental data previously extracted. During FEES video-audio recordings, the two examiners minimized verbal comments related to clinical findings, limiting them to standardized indications such as the initiation of bolus intake, specified by consistency and volume. Verbal cues and requests for compensatory strategies were retained in the recordings, as they constitute an integral part of DOSS-based swallowing assessment. At the end of the examination, each patient was assigned to a specific DOSS category.

The comprehensive dysphagia evaluation integrated with FEES served as reference for analyzing the study results. Four food consistencies were tested, according to International Dysphagia Diet Standardization Initiative (IDDS) standards: mildly thick (2), extremely thick (4), easy to chew (7), and thin liquid without thickener (0). For all four consistencies, diluted methylene blue was used as a biological tracer, for bolus volumes of 5 ml and 10 ml, for a total of at least three swallowing trials for each bolus tested, with a standard sequence: extremely thick, mildly thick, thin liquid, and easy to chew.

For the purposes of the present study (Table 1), DOSS levels 7 − 6 and 5–2 respectively defined a safe swallowing (SS) and an unsafe swallowing (US), while the Yale Pharyngeal Residue Severity Rating Scale [25] was used to classify the extent of post-swallowing residue.

**Table 1:** DOSS (reworked) showing its sub-sections (nutritional status, independence and functional status)

DOSS Level	SS		US				
	Level 7 Normal diet	Level 6 Within functional limits	Level 5 Mild dysphagia	Level 4 Mild/Moderate dysphagia	Level 3 Moderate dysphagia	Level 2 Moderately severe dysphagia	Level 1 Severe dysphagia
Nutritional status	Full per-oral nutrition	Full per-oral nutrition. May need extra time for meal	May need one consistency restricted	One or two consistencies restricted	Two or more consistencies restricted.	Two or more consistencies restricted or one consistency safely. Partial oral nutrition	Unable to tolerate any P.O. safely
Independence	Normal	Normal/ Modified	Distant supervision	Intermittent supervision/ cueing	Total assist, supervision, or strategies	Maximum assistance and use of strategies	Non oral nutrition N.P.O.
Functional status	No aspiration or penetration	Mild oral or pharyngeal delay and/or retention spontaneously compensated	Penetration and/or aspiration with at least one consistency. Reflexive valid cough. Mild oral and/or pharyngeal retention spontaneously cleared	Penetration with two consistencies. Aspiration with one consistency. Weak or absent reflexive cough. Mild oral and/or pharyngeal retention cleared with cue	Penetration or aspiration with two or more consistencies. Weak or absent reflexive cough. Moderate oral and/or pharyngeal retention cleared with cue	Aspiration and penetration with two or more consistencies with no reflexive cough. Severe oral and/or pharyngeal retention, unable to clear or cleared with multiple cues	Silent aspiration with two or more consistencies. Unable to achieve swallow. Severe retention in pharynx, unable to clear

Abbreviations: *SS *safe swallowing, *US *unsafe swallowing. For details, see reference [5]

Three ENT specialists with at least three years of experience in clinical assessment of heterogeneous dysphagias completed a structured training program, consisting of two 4-hour sessions conducted by the two experienced examiners previously involved in the comprehensive dysphagia evaluation. The training focused on the application of FEES and DOSS in the assessment of neuromuscular origin dysphagia. The three specialists reviewed 30 FEES video-audio recordings selected from the case series, projected on a 40-inch HD screen, blinded to results of the comprehensive clinical dysphagia evaluation. Raters were provided with information on the medical diagnosis and independently assigned a DOSS level to each case. To assess intra-rater reliability, the 30 FEES video-audio recordings evaluation was repeated after 14 days. At the request of one or more raters, recordings could be viewed a second time. The 30 FEES video-audio recordings sample included cases representative of all DOSS levels. Specifically, seven cases with DOSS level 7 and eight cases with level 6 were randomly selected from the SS group, while among US patients one case with DOSS level 2, two cases with level 3, five cases with level 4, and seven cases with level 5 were included.

### Non-instrumental measures

Patients reported measures (PRMs), collected by a neurologist certified in the ALSFRS-R questionnaire and a speech therapist experienced in swallowing disorders two to three days before the comprehensive dysphagia evaluation, included I-3 from ALSFRS-R questionnaire [10] and EAT-10 [13]. I-3 consists of five options indicating the functional status of swallowing (4 = normal eating habits; 3 = early eating problems, occasional choking; 2 = dietary consistency changes; 1 = needs supplemental tube feeding; 0 = NPO). EAT-10 offers ten items scored on a 5-point scale (0 = no problem to 4 = severe problem), referring to the patient’s perception of specific swallowing difficulties.

All patients underwent selected NICMs extraction on the same day as the comprehensive dysphagia evaluation. Grading of Hyo-Laryngeal Excursion (HLE), quality of Voluntary Cough (VC), quality of Voice and onset of Reflex Cough and/or throat clearing after swallowing (VRC) was based on Mann Assessment of Swallowing Ability [26], with scores ranging from 1 to 4 (Table 2). VC and VRC were assessed by three trained speech pathologists with different levels of experience in neurologically based swallowing disorders (8 years for the most experienced rater and 5 years for the other two). Two separate evaluations were conducted 14–21 days apart, based on listening to audio recordings (Voice Analysis Module, Daisy 3.6.13: Biomedica, Amplifon; Milan - Italy), extracted through a professional microphone (Sennheiser microphone e835S; Sennheiser Electronics. Wedemark, Germany) and reproduced using high-fidelity speakers. (PreSonus Eris E3.5 2nd Gen. Thomann, Italy). Each rater was asked to formulate an independent judgment after listening to each individual audio recording presented in random order, without any data that could bias the evaluation, and with no time limit. HLE was graded by two of the three trained speech pathologists recruited for the study, with 8 and 5 years of experience in swallowing disorders respectively, using the manual palpation method [27], asking each patient to swallow saliva at least three times. For maximum phonation time (MPT), a cut-off of 10 s was used as the minimum threshold indicative of normality [20], extracting the longest sample from three trials of sustained phonation of vowel /a/ for each patient. The Voice Analysis Module of the Daisy 3.6.13 program was used for this measurement. MPT was graded (Table 2) on a scale from 1 to 4 (1 = ≥ 10 s.; 2–4 = < 10 s.).

**Table 2:** Grading of VC, MPT, HLE and VRC employed in the study

Measures	Score				
	1		2	3	4
VC	No abnormality		Cough attempted but is hoarse in quality	Attempt inadequate Incomplete attempt	No attempt
MPT	*≥* 10 s.		< 10 - *≥* 5 s.	< 5 - *≥*1 s.	< 1 s.
HLE	Immediate & Complete		Mildly restricted	Incomplete	Absent, no swallow initiated
VRC	No abnormality	Coughing (and/or throat clearing)		Gurgling (and/or wet voice)	Unable to perform

Abbreviations: *VC* voluntary cough, *MPT* max. phonation time, *HLE* hyolaryngeal excursion, Voice & Reflex Cough (VRC)

### Statistical analysis

Continuous variables are reported as mean ± standard deviation (SD) for normally distributed variables and as median (interquartile range) for non-normally distributed variables, while categorical variables are expressed as absolute numbers and percentages. Comparisons between patients with safe swallowing (SS) and unsafe swallowing (US), as defined by the DOSS, were performed using independent samples t-tests for normally distributed continuous variables, Mann–Whitney U tests for non-normally distributed continuous variables, and χ² tests for categorical variables, as appropriate.

Inter-rater and intra-rater reliability for NICMs, including VC, VRC, and HLE, were assessed using Cohen’s weighted Kappa for intra-rater agreement and Fleiss’ Kappa for inter-rater agreement. A Kappa value ≥ 0.70 was considered indicative of satisfactory agreement.

To explore the ability of PRMs (I-3 and EAT-10) and NICMs to identify patients with unsafe swallowing, receiver operating characteristic (ROC) curve analysis was performed using DOSS-based classification (SS vs. US) as the binary reference standard. For each variable, the area under the curve (AUC), sensitivity, specificity, positive predictive value (PPV), negative predictive value (NPV), positive likelihood ratio (LR+), and negative likelihood ratio (LR–) were calculated. Optimal cut-off values were identified using the Youden index.

A composite clinical score, termed the Composite Clinical Features Index (CCFI), was constructed by combining selected NICMs showing the strongest discriminative contribution with respect to swallowing safety. The relative contribution of each variable to the composite score was estimated using multiple linear regression analysis as a weighting procedure. The CCFI was treated as a continuous score, and its discriminative performance in distinguishing SS from US patients was evaluated exclusively by ROC curve analysis. All statistical analyses were performed using SPSS software (version 13.0; SPSS Inc., Chicago, IL, USA). A two-tailed *p* value < 0.05 was considered statistically significant.

## Results

### Distribution of participants in DOSS levels

Eighty-seven out of 113 patients (77.0%) exhibited a SS for consistencies and bolus volumes tested during FEES. Thirty-three patients/87 (37.9%) were classified at DOSS level 7, indicating normal swallowing, 54/87 (62.1%) were at level 6, reflecting swallowing “within functional limits”. Twenty-six patients (23.0%) showed an US, due to penetration-aspiration events and/or pharyngeal residue involving at least one consistency. Among these, eighteen (69.2%) were assigned DOSS level 5, five (19.2%) level 4, two (7.7%) level 3, and one (3.8%) level 2. Patients with US received restriction, up to exclusion, of one or more consistencies, with variable supervision during meals. The DOSS level 2 patient, who could safely manage only one consistency with total assistance from a caregiver, was recommended an artificial nutritional support.

### Inter- and intra-rater reliability of DOSS ratings

Inter- and intra-rater agreement for DOSS ratings was excellent across all raters and assessment sessions (κ > 0.90). Rater 1 demonstrated almost perfect intra-rater agreement (κ = 0.916, *p* < 0.001), rater 2 showed perfect intra-rater reliability, with complete agreement between the two rating sessions (κ = 1.000, *p* < 0.001), rater 3 also demonstrated almost perfect intra-rater agreement (κ = 0.914, *p* < 0.001). Percent exact agreement (PEA) between sessions was high for all raters, ranging from 93.3% to 100%. Rater 2 showed complete agreement, whereas raters 1 and 3 differed in only two cases each. Percent close agreement (PCA), defined as agreement within ± 1 DOSS level, was 100% for all raters, indicating that all discrepancies were limited to a single-level difference.

### NICMs intra and inter-rater reliability

Intra-rater reliability showed excellent agreement for both voluntary cough (VC: κ = 0.96, 0.92, 0.93) and reflexive cough/throat clearing (VRC: κ = 0.97, 0.89, 0.92). Inter-rater reliability also showed strong consistency across raters: for VC, κ = 0.852 at the first evaluation (T1) and κ = 0.877 at the second (T2); for VRC, κ = 0.962 at T1 and κ = 0.906 at T2. For HLE, the inter-rater agreement was κ = 0.89. To ensure consistency, the most experienced rater served as reference for all NICMs.

### Demographic data and comparison between SS and US groups

The participants mean age was 64 years, with no significant difference between the SS and US groups. Male participants were significantly more numerous than females. A highly significant difference was observed in the distribution of spinal versus bulbar onset, with spinal onset being more prevalent overall. This difference remained statistically significant when comparing the SS and US groups (*p* < 0.05), with bulbar onset more frequent in the US group and spinal onset more common in the SS group. The average diagnostic delay was 15.8 months, with no significant difference between groups. The overall ALSFRS-R score differed significantly between the SS and US groups (*p* < 0.05), being lower in the US group. However, no significant difference was found in the ALSFRS-R progression rate between the two groups (Table 3).

**Table 3:** Demographic data and clinical measures in the study patients

	Overall(*n* = 113)	SS(87; 76.9%)	US(26; 23.1%)	*P*
Age (years)	64.0 (11.4)	63.6 (12.0)	65.4 (9.6)	0.219
Gender	73 males (64.6%) 40 females (35.4%)	53 males (72.6%) 34 females (85.0%)	20 males (27.4%) 6 females (15.0%)	0.002
Onset	86/113 spinal (76.1%) 27/113 bulbar (23.9%)	70/87 spinal (80.5%) 17/87 bulbar (19.5%)	16/26 spinal (61.5%) 10/26 bulbar (38.5%)	< 0.001 < 0.001 0.047
Diagnostic Delay (months)	11 (7–20)	11 (7–19)	11.5 (7-23.5)	0.735
ALSFRS-R	36 (30–41)	37 (31–41)	32 (25.75-36)	0.024
ALSFRS-R progression rate	0.83 (0.52–1.9)	0.8 (0.51–1.81)	1.04 (0.6–2.5)	0.780
I-3 ALSFRS R	3.21 (0.9)	3.39 (0.8)	2.62 (0.9)	< 0.001
Eat-10	7.04 (8.3)	5.02 (6.8)	13.8 (9.3)	< 0.001
VC	1.43 (0.74)	1.26 (0.51)	2.0 (1.05)	< 0.001
HLE	1.63 (0.68)	1.54 (0.66)	1.92 (0.69)	0.008
VRC	1.22 (0.50)	1.10 (0.31)	1.62 (0.75)	0.002
MPT (s)	12.2 (5.3)	13.21 (4.96)	8.88 (4.94)	< 0.001

Abbreviations: *SS* safe swallowing, *US* unsafe swallowing, *I-3* item 3 ALS FRS-R, *VC* voluntary cough, *HLE* hyo-laryngeal excursion, *VRC* voice and reflex cough, *MPT* maximum phonation time. Data are presented as mean ± SD, or median (interquartile range)

**Fig. 1:**
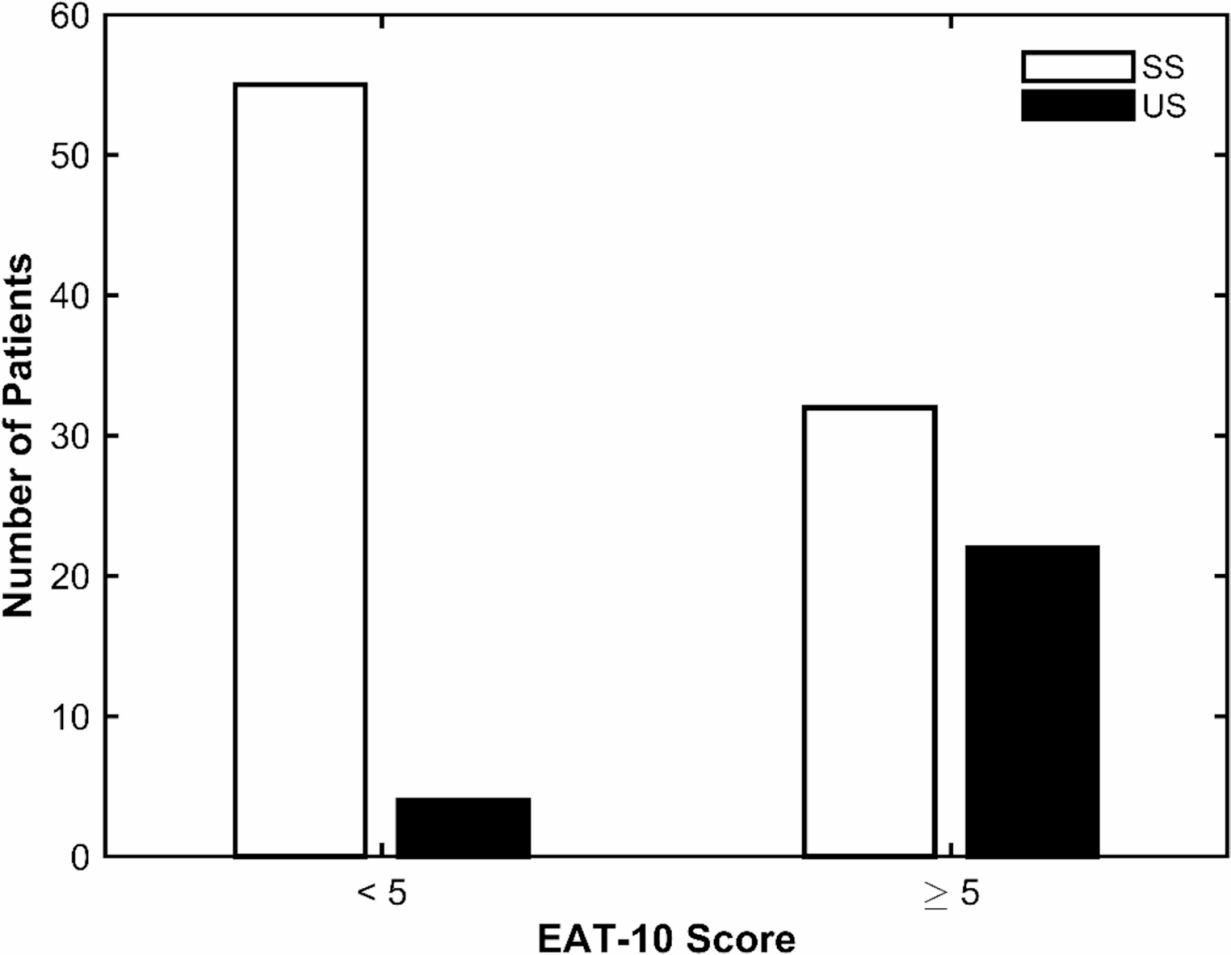
Distribution of study patients for safe (SS) and unsafe (US) swallowing, as defined by DOSS, according to EAT-10 scores (cut-off = 5)

**Fig. 2:**
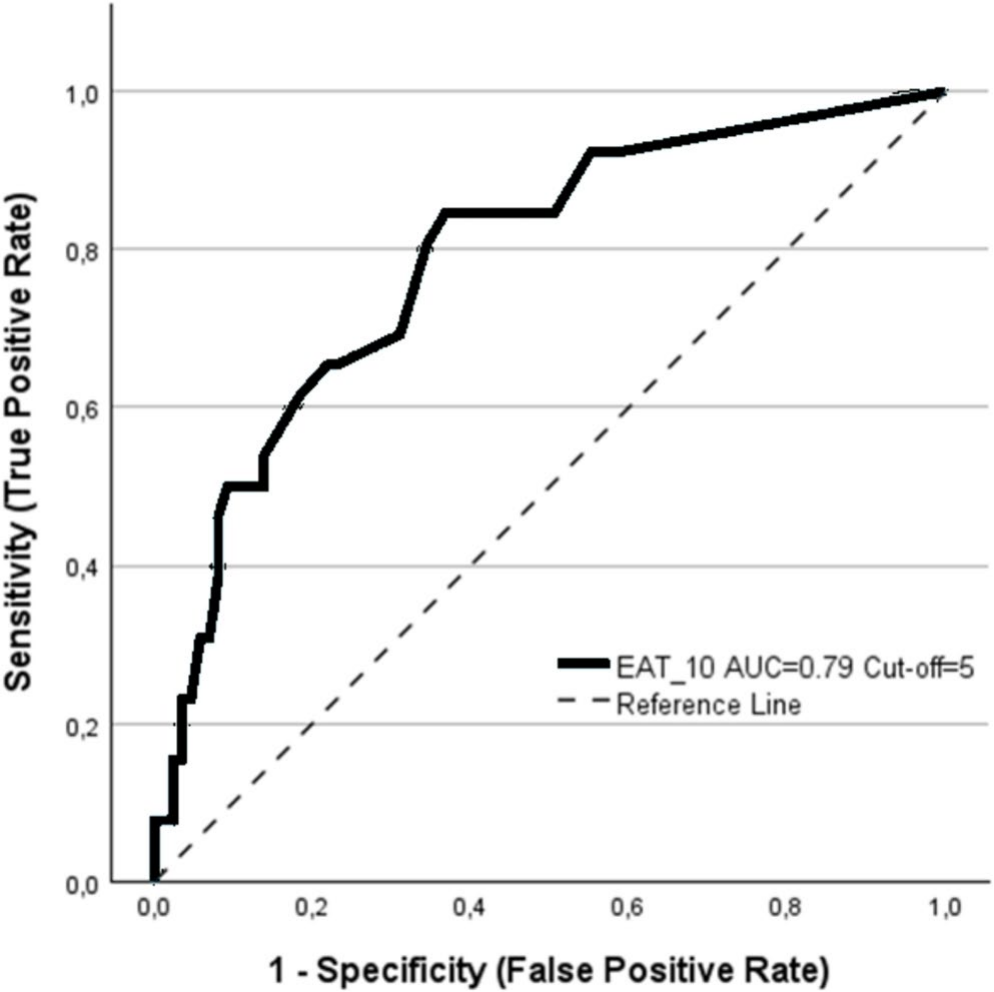
ROC curve of EAT-10 for discriminating safe (SS) versus unsafe (US) swallowing, as defined by DOSS (AUC = 0.790)

**Fig. 3:**
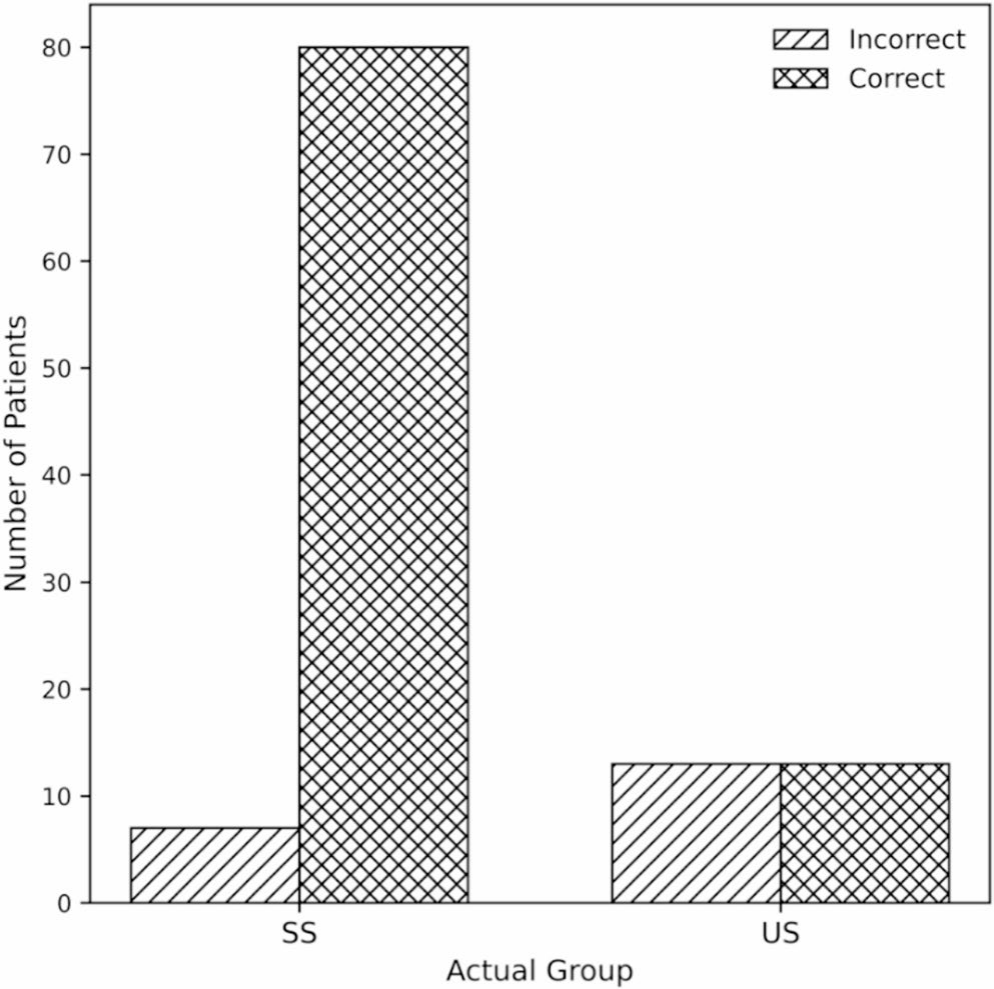
Distribution of Composite Clinical Features Index (CCFI) values in safe (SS) and unsafe (US) swallowing groups

**Fig. 4:**
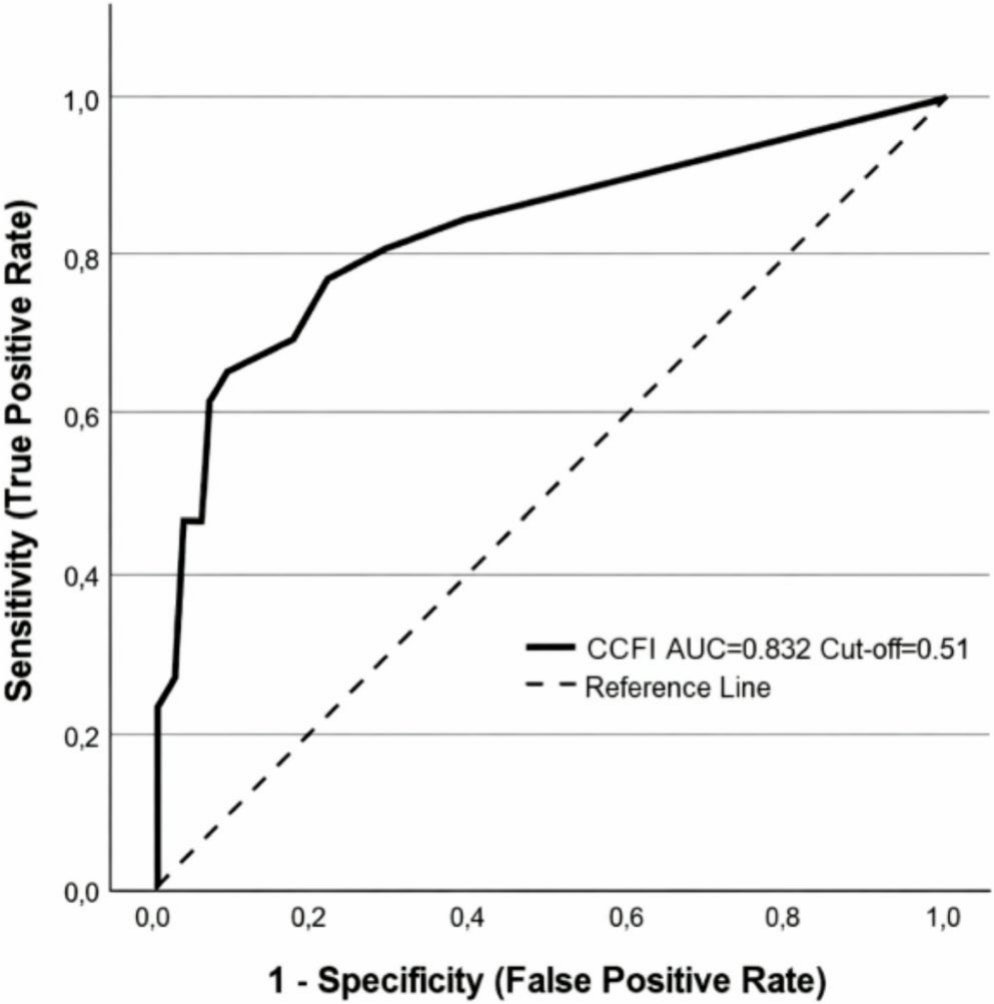
ROC curve of Composite Clinical Features Index (CCFI) discriminating safe (SS) versus unsafe (US) swallowing, as defined by DOSS (AUC = 0.832)

Highly significant differences (*p* < 0.001) were observed in PRMs, specifically the I-3 and EAT-10 scores, between the SS and US groups. Similarly, statistically significant or highly significant differences were found between the two groups for HLE (*p* = 0.008), VC (*p* < 0.001), VRC (*p* = 0.002), and MPT (*p* < 0.001). Table 4 summarizes the diagnostic performance of PRMs and three NICMs -VRC, VC, and MPT- selected based on their higher discriminative contribution (VRC: 1.492; VC: 0.899; MPT: 0.650). The CCFI, derived as a weighted linear combination of VC, VRC, and MPT, was defined as follows: CCFI = − 0.627 + 0.258⋅VC + 0.267⋅VRC + 0.087⋅MPTbin. Both EAT-10 (Figs. 1, 2) and CCFI (Figs. 3, 4) demonstrated an AUC > 0.70.

**Table 4:** Diagnostic performance of PRMs and NICMs

CUT-OFF	I-3	EAT-10	VRC	MPT	CCFI
	3	5	2	2	0.51*
AUC	0.689	0.790	0.691	0.693	0.832
SENS	76.9%	84.6%	46.2%	61.5%	50.0%
SPEC	60.9%	63.2%	89.7%	77.0%	92.0%
PPV	37.0%	40.7%	64.7%	44.4%	65.0%
NPV	89.8%	93.2%	89.1%	87.0%	86.0%
LR +	1.97	2.30	6.13	2.67	6.21
LR -	0.38	0.24	0.41	0.50	0.54

Abbreviations: *AUC* area under the curve, *SENS* sensitivity, *SPEC* specificity, *PPV* positive predictive value, *NPV *negative predictive value, *LR +* likelihood ratio +, *LR* - likelihood ratio -, *I-3* Item 3 ALS FRS-R, *VRC* voice and reflex cough, *MPT* maximum, phonation time, *CCFI* composite clinical features index. * Derived from Youden index

The AUC for I-3 and the other non-instrumental clinical measures was below the 0.70 threshold. The highest sensitivity values were observed by EAT-10 (84.6%) and I-3 (76.9%). In terms of specificity, the best diagnostic performance was demonstrated by CCFI (90.8%), followed by two individually analyzed clinical variables: VRC (89.7%) and MPT (77.0%). The highest positive predictive values were achieved by CCFI (68.0%) and VRC (64.7%). All variables analyzed yielded negative predictive values above 85%, with the highest value observed for EAT-10 (93.2%). The most favorable values for positive likelihood ratios were obtained by CCFI (6.21) and VRC (6.13). EAT-10 also demonstrated the best performance in terms of negative likelihood ratio (0.24).

## Discussion

People with ALS may experience severe swallowing disorders, which put the lower airways at risk of invasion and make swallowing poorly effective and unsafe [15, 19]. In the present study, comprehensive dysphagia clinical data together with instrumental findings obtained by FEES in 113 patients were distributed according to DOSS.

To date, to the best of our knowledge, the present investigation represents the first validity and reliability study in which DOSS was used with FEES in a large sample of ALS patients. The main criteria followed by DOSS for the definition of swallowing safety consist in detection of airways food invasion and efficiency of reflex reactions, as well as in quantification of post-swallowing residue and ability of its spontaneous cleansing, for pre-established bolus consistencies and volumes [5–7]. Fransson et al. recently reported a high level of agreement between DOSS and Functional Oral Intake Scale, based on concordant ratings of voiceless FEES video recordings by eleven speech pathologists blinded to diagnosis and additional clinical information in 11 heterogeneous dysphagic patients and four healthy adults [7]. In the same study, inter- and intra-rater reliability of DOSS ratings were found to be almost perfect, in line with the results of the original work by O’Neil and colleagues, who evaluated 135 patients with heterogeneous dysphagia undergoing comprehensive clinical assessment with VFSS [5]. Taken together, these studies reported similarly high inter- and intra-rater agreement for DOSS classification across different dysphagic populations, despite differences in instrumental techniques (VFSS vs. FEES) and in the modality of case presentation to raters.

In the present study, inter- and intra-rater agreement for DOSS ratings was excellent across all raters and assessment sessions (κ > 0.90).These findings are consistent with previous data obtained in heterogeneous dysphagias [5, 7] and support the reliability of DOSS for classifying swallowing safety in ALS. The original article by O’Neil et al. [5] highlighted that DOSS requires the integration of multiple clinical factors when assigning scale levels, such as the amount of supervision and the patient’s current medical status. Furthermore, as specified by Fransson et al. [7], an accurate DOSS scoring should necessarily be based on identification of compensatory strategies by the patient, recognition of acoustic–perceptual voice changes during the examination, and evaluation of the patient’s ability to respond to verbal cues provided by the examiner. The lack of one or more of these clinical findings may increase judgmental difficulty for raters and result in reduced agreement in DOSS scoring, as reported by Zarkada and Regan in their analysis of DOSS and VFSS video clips from patients with heterogeneous dysphagia [28].

In the present series, 77% of the study cases were classified in SS group (DOSS levels 7 − 6). In 23% of the study cases, FEES demonstrated an US (DOSS levels *≤* 5), up to detection of a single consistency safely swallowed. Spinal onset was more prevalent than bulbar onset in the study patients and US more frequent in bulbar-onset cases than in those with spinal onset. ALSFRS-R scores were also significantly lower in patients with US compared to those with SS. These findings agree with data by Onesti and colleagues [29], who identified an US in about 95% of bulbar-onset cases at the start of follow-up in a retrospective longitudinal study of 145 patients assessed by FEES. In the same study, ALSFRS-R and its bulbar subscale (items 1–3) scores resulted significantly lower in dysphagic patients than in non-dysphagic ones at baseline. Likewise, Xia et al. [11], in a study involving 68 patients, reported an US in eight/13 bulbar-onset patients (61.5%), with significantly ALSFRS-R bulbar subscale lower scores.

Differences between our study and previous researches, with respect to the absolute number of ALS patients with dysphagia and the spinal-bulbar onset ratio, may arise from dissimilar recruitment criteria as well as baseline tests and endpoints used to define dysphagia [11, 29]. In studies known to us, swallowing impairment in ALS patients was graded according to PAS with dysphagia endpoints scoring > 1 [29] or > 2 [11, 14]. To overcome the PAS limitation, that does not rate pharyngeal residue, some studies added tools to assess post-swallow residue, such as the P-score [29, 30], or integrated scales like the Dynamic Imaging Grade of Swallowing Toxicity [12] to obtain a single classification that could better reflect the overall swallowing function. In all these studies [11, 12, 14, 29], the worst PAS score was used to stratify the sample into SS and US categories or to further distinguish aspiration from penetration, typically adopting a cut-off PAS score *≥* 6.

In our opinion, DOSS does not compete with PAS, but complements it by translating impairment into clinically actionable nutritional decisions. PAS worst score criterion is apparently precise in defining samples categorically ordered by severity of dysphagia, proving not sufficiently informative in a clinical context [31, 32].

In our study, PRMs (EAT-10, I-3) and NICMs (HLE, VC, VRC, and MPT) showed highly significant differences between the DOSS-based SS and US groups (Table 3). EAT-10 and CCFI achieved an AUC of at least 0.70, supporting acceptable criterion validity [33]. In the present cohort, an EAT-10 cut-off score of 5 (Figs. 1, 2) demonstrated high sensitivity for identifying patients with US (84.6%) and high negative predictive values (93.2%). However, it was also associated with a relatively high proportion of false positives, as indicated by a specificity of 63.2%, which in turn reduced the positive predictive value (40.7%).

Similar results were observed in other studies, with reference to an EAT-10 cut-off of 3, based on PAS endpoints *≥* 3 for the definition of US obtained by VFSS [11, 14]. Xia et al. [11], in particular, demonstrated an AUC of 0.87 for EAT-10 in a study of 68 ALS patients and found no substantial differences in discriminative ability compared to other swallowing screening tools (ALSFRS bulbar subscale, Water-Swallowing Test, Sydney Swallow Questionnaire). The diagnostic utility of ALSFRS-R bulbar subscale as well as of I-3, has also been investigated by Chapin and colleagues [12] in a series of 197 ALS patients in which the endpoints were conventionally defined by PAS *≥* 3 (169 US cases and 28 SS cases) and by DIGEST scale (81 normal swallowing cases and 116 dysphagic cases). These authors expressed skepticism about these screening tools performance, despite an AUC greater than 0.70 for ALSFRS bulbar subscale with both scales used (PAS and DIGEST) and for I-3 for PAS, with sensitivity and specificity of just under 80% and just over 60% respectively for both measures.

Regarding the NICMs examined in the present study (HLE, VC, VRC and MPT), none of them individually considered showed a sufficient discriminating capacity in distinguishing SS from US cases. However, VRC, VC and MPT were selected based on their individual discriminative performance, contributing to the definition of a composite discriminative index (CCFI) with high overall discrimination (AUC = 0.832). Such features have been included in screening tools or clinical evaluations following acute stroke for many years [17, 18, 26] and more recently used as part of reference tests to verify the accuracy of other screening tools in the same clinical setting [34]. The diagnostic outcome of the CCFI in the present study (Figs. 3, 4) is due to high specificity (92%) and negative predictive value (86.0%), despite a lower sensitivity (50%). These results, in our opinion, do not compromise the clinical relevance of clustered clinical signs when interpreted by experienced clinicians. In this regard, Warms and Richards reported how a single clinical feature is insufficient to reliably predict an airways food invasion, concluding that only a cluster of clinical signs can alert clinicians to penetration/aspiration risk [35]. On the other hand, Plowman et al. [36] showed the relevance of quality of voluntary cough as a single screening tool in ALS in 70 patients tested by spirometry and VFSS. At least three of the spirometric measures used in this study (cough volume acceleration, peak expiratory flow rate, peak expiratory flow rise time) clearly differentiated SS condition from US condition (endpoint PAS *≥* 3), with an AUC of 0.85 for cough volume acceleration associated with a sensitivity and specificity of 91.3% and 82.2%, respectively. These favorable results are counterbalanced by a low clinical feasibility and higher costs, as well as lack of standardization regarding low-cost portable instruments [37]. As previously reported, in the present study MPT also significantly differentiated SS and US groups. Nevertheless, MPT demonstrated an insufficient discrimination power between the two groups as a single measure, with an AUC of 0.693, a sensitivity of just over 60% and a specificity of 77%, in partial agreement with other findings obtained in different clinical populations [23, 38].

Data on the diagnostic reliability of non-instrumental measures for dysphagia screening in ALS patients should be carefully evaluated also considering disease and concomitant dysphagia progression [29, 39]. In this regard, Onesti et al. [29] found a PAS score > 2 in approximately 59% and 83% of the study cases, respectively at the beginning and at the end of the follow-up, which occurred on average after 20 months, with a dysphagia incidence higher than 94% in bulbar onset cases. Likewise, Mariani and colleagues [39] in a retrospectively studied series of 108 ALS patients found a PAS score > 2 in 47.5% of study cases at the first FEES examination, with penetration at T0 in up to 85% of cases with bulbar onset and disease progression rate > 0.5.

The potential dysphagia rapid progression in people with ALS raises questions about the usefulness of screening tools, which must address the need for pragmatism in managing a triage flow in a busy ALS clinical setting, regardless of their accuracy. In this respect, PRMs are in principle endowed with a greater clinical feasibility than NICMs, and also in our study EAT-10 demonstrated a considerable diagnostic performance. However, PRMs are burdened by intrinsic limitations, as they can be technically controversial from a psychometric point of view and questionable in patients with cognitive and emotional disorders [8, 40].

We cannot ignore some significant limitations of our study. Its cross-sectional design does not allow evaluation of the DOSS role in monitoring dysphagia over the course of ALS progression. Moreover, the pathophysiological mechanisms underlying swallowing disorders were beyond the scope of the present study and were not investigated, although they represent relevant targets for future research.

In conclusion, the DOSS accurately defined ALS patients’ nutritional profile in this cohort, providing clinically meaningful indications regarding dietary restrictions, the need for supervision during meals, and appropriate nutritional strategies. The observed concordance between comprehensive dysphagia evaluation and independent rater judgments indicates high DOSS reliability and supports its use in the assessment of dysphagia in ALS. Non-instrumental measures identified in the present study should be interpreted with extreme caution when used for screening purposes and are best applied within structured triage systems for the management of complex clinical scenarios, taking into account their inherent limitations and the rapid progression of dysphagia in ALS.

## Data Availability

The datasets generated and analysed during the current study are available from the corresponding author on reasonable request.
